# Bibliometric Analysis of Malaysian Orthopaedic Journal using Scopus Database

**DOI:** 10.5704/MOJ.2407.001

**Published:** 2024-07

**Authors:** RY Kow, CL Low, AA Abbas, AH Zulkifly

**Affiliations:** 1 Department of Orthopaedics, Traumatology and Rehabilitation, International Islamic University Malaysia, Kuantan, Malaysia; 2 Department of Radiology, International Islamic University Malaysia, Kuantan, Malaysia; 3 National Orthopaedic Centre of Excellence for Research and Learning (NOCERAL), Department of Orthopaedic Surgery, Universiti Malaya, Kuala Lumpur, Malaysia

**Keywords:** bibliometric analysis, Malaysia, orthopaedic, journal

## Abstract

**Introduction:**

The Malaysian Orthopaedic Journal (MOJ) (ISSN 1985-2533 / 2232-111X) is the official publication of the Malaysian Orthopaedic Association (MOA) and the ASEAN Orthopaedic Association (AOA). In May 2007, MOA published the first standalone issue of MOJ with the aim of disseminating new knowledge and providing updates in orthopaedics, trauma and musculoskeletal research. Since then, MOJ has grown significantly, achieving indexing in numerous databases and attaining a 2nd Quartile (Q2) rank in the Scopus database in 2022. This bibliometric analysis aims to explore the trends and distribution of articles published in MOJ.

**Materials and Methods:**

Bibliometric data for MOJ was extracted from the SCOPUS database, covering the years from its indexing to 2022. Information such as authors, country, document type, author’s keywords, citations, and other parameters were extracted using the bibliometrix package in the R Studio software. The data were then presented in tables and illustrative graphs using the same software.

**Results:**

A total of 305 articles were retrieved from the Scopus database during the study period. Two-thirds of the articles were original articles and review articles. The highest number of citations received by an article is 56, and top ten articles in MOJ were authored by researchers from seven different countries, highlighting the journal’s diversity. Despite receiving submissions from various countries, there is minimal collaboration between authors of different countries. Keywords such as “covid-19” and “pandemic” dominate the authors’ keyword section due to the once-in-a-life-time COVID-19 which during the study period, resulting in numerous publications related to this issue.

**Conclusion:**

This bibliometric analysis reviews all the articles indexed in the Scopus database and provides insight into the contributors’ information and the trends in orthopaedic research. By identifying the lack of collaboration between countries, it is hoped that this analysis can inspire more orthopaedic surgeons and researchers to collaborate and produce high-quality publications.

## Introduction

The Malaysian Orthopaedic Journal (MOJ) (ISSN 1985-2533 / 2232-111X) is the official publication of the Malaysian Orthopaedic Association (MOA) and the ASEAN Orthopaedic Association (AOA)^1,2^. Currently published triannually, MOJ is normally released in March, July, and November each year. In May 2007, MOA published the first issue of MOJ with the aim of disseminating new knowledge and provide updates in Orthopaedics, trauma and musculoskeletal research. MOJ remained a biannual publication from 2007 to 2009. Starting in 2010, due to an increasing number of submissions and accepted articles, the editorial board decided to increase the publication frequency to triannually, a practice that continues to this day. During that time, the first issue of MOJ each year (the March issue) is dedicated to serving as the ASEAN edition of MOJ, where it serves as a continuation of the ASEAN Orthopaedic Journal.

As an open-access publication, readers can access all the published MOJ articles free of charge. They are free to use, share, or adapt the material published in MOJ without any fees, as long as they properly acknowledged or attribute the authors, following the Creative Commons license (CC BY 4.0). MOJ is currently indexed in a myriad of databases, including SCOPUS, Emerging Sources Citation Index (ESCI), Pubmed, Pubmed Central, DOAJ, MyCite, and others. Being indexed in these renown databases demonstrates the recognition MOJ rightfully deserves. In addition, it serves as a benchmark for monitoring the journal’s overall progress. For example, since being indexed in the SCOPUS database in 2015, MOJ’s ranking has improved from the 4th Quartile (Q4) in 2016 to the 2nd Quartile (Q2) in 20223.

Bibliometric analysis is an excellent tool to gauge the scholarly impact of scientific publications. When coupled with a database like SCOPUS, a bibliometric analysis can reveal the scientific impact of published articles in MOJ and their authors through indicators such as citations and the number of publications. Besides that, bibliometric analysis can aid in outlining the research interests, demarcating emerging trends, and identifying collaboration patterns among authors. In some instances, it also helps to identify under-explored areas of research topic, where the researchers and institutions can focus their efforts. This review aims to perform a bibliometric analysis on MOJ using the SCOPUS database.

## Materials and Methods

The bibliometric data for MOJ was extracted from the SCOPUS database, covering the years from its indexing to 2022. This extracted data was imported into R Studio 2021 for Windows with the bibliometrix package installed in R, and Microsoft Excel 2019 [Microsoft ^®^ Corp., Redmond, WA]^4,5^. Information such as authors, country, document type, authors’ keywords, citations and other parameters were extracted using the bibliometrix package in the R Studio software^4-5^. The data were then presented in table and illustrative graphs using the same software. The whole process was cross-checked by the second author to avoid any selection bias.

## Results

A total of 305 articles were retrieved from the SCOPUS database for the years 2015 to 2022. Table I summarises the type of articles published in MOJ during the study period. More than half of the articles are original articles (n=166; 54.4%). This is followed by case reports (n=91; 29.9%), special articles (n=18; 5.9%), letters to editor (n=21; 6.9%), quizzes (n=5; 1.6%), erratum (n=3; 1.0%), and editorial (n=1; 0.3%).

**Table I:** The type of published articles in MOJ from the year 2015 to year 2022.

Article type	Number	Percentage
Special article	18	5.9
Original article	166	54.4
Case report	91	29.9
Letter to editor	21	6.9
Erratum	3	1.0
Editorial	1	0.3
Quiz	5	1.6
Total	305	100

Among the 305 published articles during the study period, each article's citations ranged from 0 to 56. Table II summarises the top 10 articles in MOJ with the highest number of citations. The article with the most citations was authored by Stewart from the United Kingdom, with a total of 56 citations^7^. This was followed by an article by Tay from Singapore (27 citations), Esa from United Kingdom and Passiatore from Italy (16 citations each), Tamburrelli from Italy (15 citations), Dhillon from Malaysia (14 citations), Soraganvi from India and Limpaphayom from Thailand (12 citations each) and Ong from Malaysia and Maniar from United States of America (11 citations each)^8-16^. All of the top 10 articles except one, were published in the years 2019 and 2020. More than half of the articles (six articles) were review articles. The other four articles were original articles, with two of them being randomised controlled studies and the other two being retrospective studies.

**Table II:** List of Top 10 Articles in MOJ.

Rank	No. of Citations	First Author	Country of First Author	Year of Publication	Article Title	Type of article
1	56	Stewart SK	United Kingdom	2019	Fracture Non-Union: A Review of Clinical Challenges and Future Research Needs^6^	Review
2	27	Tay K	Singapore	2020	COVID-19 in Singapore and Malaysia: Rising to the Challenges of Orthopaedic (Practice in an Evolving Pandemic^7^	Review Special article - COVID-19)
3	16	Esa A	United Kingdom	2019	Extracellular Vesicles in the Synovial Joint: Is there a Role in the Pathophysiology of Osteoarthritis?^8^	Review
4	16	Passiatore M	Italy	2020	The Use of Alfa-Lipoic Acid-R (ALA-R) in Patients with Mild-Moderate Carpal Tunnel Syndrome: A Randomised Controlled Open Label Prospective Study^9^	Original Article (Randomised controlled study)
5	15	Tamburrelli FC	Italy	2019	The Feasibility of Long-Segment Fluoroscopy-guided Percutaneous Thoracic Spine Pedicle Screw Fixation, and the Outcome at Two-year Follow-up^10^	Original Article (Retrospective study)
6	14	Dhillon KS	Malaysia	2019	A Musculoskeletal Disorder or a Medical Myth?^11^	Review
7	12	Soraganvi P	India	2019	Is Platelet-rich Plasma Injection more Effective than Steroid Injection in the Treatment of Chronic Plantar Fasciitis in Achieving Long-term Relief?^12^	Original Article (Randomised controlled study)
8	12	Limpaphayom N	Thailand	2019	Factors Related to Early Recurrence of Idiopathic Clubfoot Post the Ponseti Method^13^	Original Article (Retrospective study)
9	11	Ong T	Malaysia	2020	The Current and Future Challenges of Hip Fracture Management in Malaysia^14^	Review
10	11	Maniar HH	United States of America	2015	The Current Role of Stem Cells in Orthopaedic Surgery^15^	Review

With regard to the author’s analysis, the top 10 authors with the most publications are summarised in Table III. Kow had the most articles published in MOJ, with a total of 11 publications. This was followed by Faisham and Low (8 publications each), Ahmad (7 publications), Mansor (6 publications), Ahmad, Hadizie, Ibrahim, and Munajat (5 publications each) and Abdul-Rashid (4 publications).

**Table III:** List of Top 10 Authors.

Rank	Author	Number of publications
1	Kow RY	11
2	Faisham WI	8
3	Low CL	8
4	Ahmad TS	7
5	Mansor A	6
6	Ahmad AR	5
7	Hadizie D	5
8	Ibrahim S	5
9	Munajat I	5
10	Abdul-Rashid AH	4

The top ten contributing institutions are summarised in Table IV. Universiti Sains Malaysia has the highest number of publications in MOJ, with a total of 81 articles attributed to it. It is followed by Universiti Malaya (55 publications), Universiti Kebangsaan Malaysia (49 publications), Singapore General Hospital (38 publications), Tan Tock Seng Hospital (24 publications), Kuala Lumpur Sports Medicine Centre (20 publications), Universiti Malaysia Sarawak (19 publications), All India Institute of Medical Sciences and Hospital Kuala Lumpur (17 publications each), and International Islamic University Malaysia (15 publications).

**Table IV:** List of Top 10 Institutions.

Rank	Institution	Number of publications
1	Universiti Sains Malaysia	81
2	Universiti Malaya	55
3	Universiti Kebangsaan Malaysia	49
4	Singapore General Hospital	38
5	Tan Tock Seng Hospital	24
6	Kuala Lumpur Sports Medicine Centre	20
7	Universiti Malaysia Sarawak	19
8	All India Institute of Medical Sciences	17
9	Hospital Kuala Lumpur	17
10	International Islamic University Malaysia	15

In terms of the corresponding author’s country, Malaysian authors contributed the most articles to MOJ, with a total of 102 publications (Table V). They are followed by authors from India (62 publications), Singapore (34 publications), Italy, Thailand and Turkey (10 publications each), United Kingdom (9 publications), Indonesia and Philippines (8 publications each) and Korea (5 publications). Further analysis reveals that most of the contributing countries have a low ratio of multiple-country publications to single-country publications. All countries except three have 10% or less of their publications consisting of multiple-country collaborations.

**Table V:** List of Top 10 contributing countries.

Rank	Corresponding Author’s Countries	SCP (%)	MCP (%)	Total
1	Malaysia	96 (94.1%)	6 (5.9%)	102
2	India	60 (96.8%)	2 (3.2%)	62
3	Singapore	32 (94.1%)	2 (5.9)	34
4	Italy	9 (90%)	1 (10%)	10
5	Thailand	8 (80%)	2 (20%)	10
6	Turkey	10 (100%)	0 (0%)	10
7	United Kingdom	7 (77.8%)	2 (22.2%)	9
8	Indonesia	8 (100%)	0 (0%)	8
9	Philippines	8 (100%)	0 (0%)	8
10	Korea	3 (60%)	2 (40%)	5

Abbreviations - SCP: single country publication, MCP: multiple countries publication

**Fig. 1:**
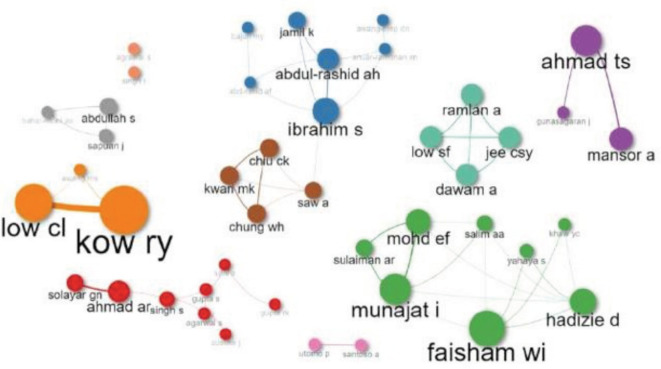
Publication parameters for co-authorship in MOJ. Different colours refer to different clusters. The collaboration between different authors is being demarcated by each cluster. Graphic illustration is produced using the open-sourced biblioshiny and bibliometrix software under the R studio^5^.

**Fig. 2:**
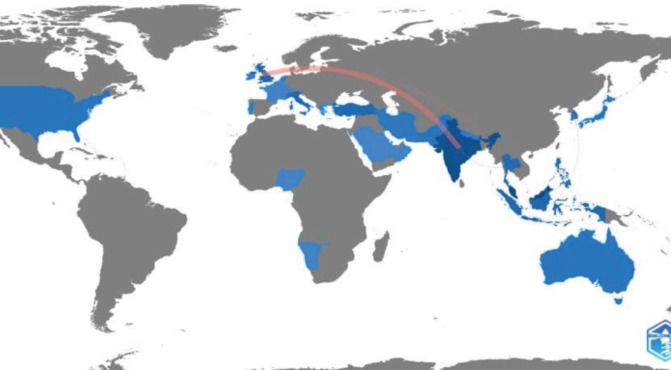
A graphical representation of the collaborations between different countries is shown. The pink lines depict collaboration between countries, while the shades of blue in each country indicate the number of publications from that country. The darker the blue colour, the more publications the country has produced in MOJ. Graphic illustration is produced using the open-sourced biblioshiny and bibliometrix software under the R studio^5^.

Fig. 1 illustrates the co-authorship of publications in MOJ. This figure portrays limited collaboration among authors from different institutions. A similar trend is observed when analysing collaboration between different countries (Fig. 2). There is limited collaboration among different countries, except for United Kingdom, India, and a few Southeast Asian countries.

**Fig. 3:**
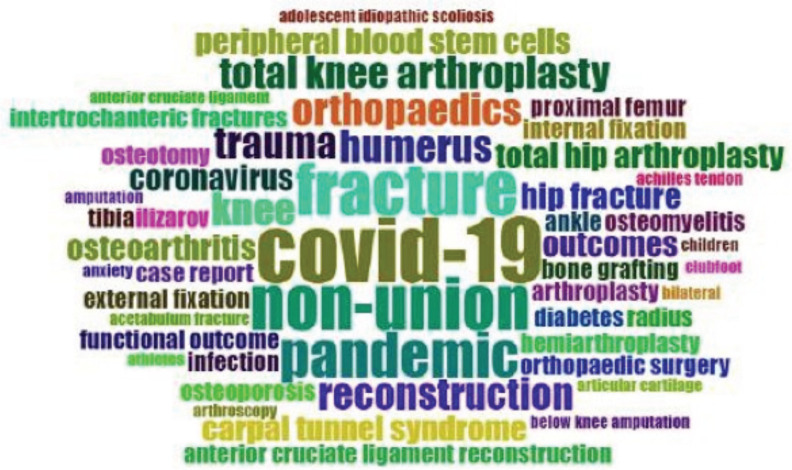
Word cloud of author’s keywords is illustrated. Word cloud is a visual representation of text data, where more frequently occurring words appearing larger and less frequent words appearing smaller. In this word cloud, the three most common terms (COVID-19, fracture and non-union) are immediately identified. Graphic illustration is produced using the open-sourced biblioshiny and bibliometrix software under the R studio^5^.

**Fig. 4:**
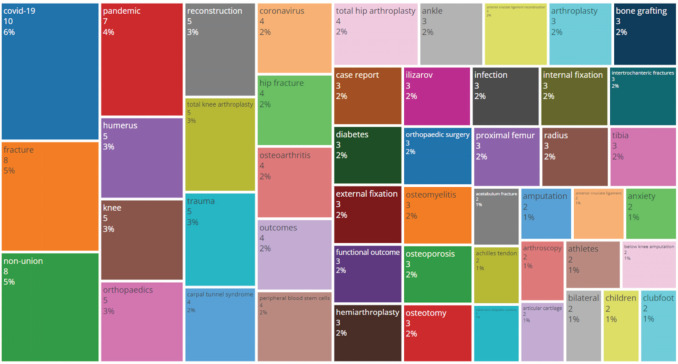
Treemap of author’s keywords is illustrated. A treemap is a type of data visualisation that displays hierarchical data in as nested rectangles in a larger rectangle. In this treemap, the three most common terms are easily identified on the left side of the rectangle. Graphic illustration is produced using the open-sourced biblioshiny and bibliometrix software under the R studio^5^.

Keywords provided by the authors are one of the important information in a journal. In this bibliometric analysis of MOJ, the top 50 commonly used author’s keywords were analysed and summarised in both word cloud format (Fig. 3) and treemap format (Fig. 4). “Covid-19” is the most common author’s keyword during the study period, where it is provided in 6% of the published articles in the MOJ. This is followed by “fracture” and “non-union” (5% each), “pandemic” (4%), “humerus”, “knee”, “orthopaedics”, “reconstruction”, “total knee arthroplasty” and “trauma” (3% each). The other author’s keywords are present in 2% or less of the published articles in the MOJ.

## Discussion

This bibliometric analysis provides insights into the trends and distribution of articles published in MOJ. The number of citations received by articles in a journal is considered one of the most important parameters for assessing the journal’s impact. Databases such as Scopus and Web of Science often categorise journals into different quartiles, with journals in the first quartile (Q1) being regarded as the best within their field, as they rank in the top 25% in terms of impact factor. These quartiles are frequently used to evaluate the standing and quality of a journal, and some institutions or funding agencies rely on information to assess the quality and impact of publications. In this context, MOJ is considered to have a moderate impact within its field, earning a Q2 ranking in the Scimago Journal and Country Rank (SJR)^3^. This means MOJ’s impact factor is higher than that of at least 50% of the other journals in the same field.

MOJ’s ranking might not be higher due to its policy of publishing a wide variety of article types. Articles such as case reports, letters to editor, quizzes, and errata are indexed in the Scopus database but often receive fewer citations. In contrast, articles like review articles and original articles, which make up two-thirds of the publications in MOJ, are frequently cited by other articles. This is evident in the fact that all of the top 10 articles with the most citations are either review articles or original articles (Table II). Rison *et al* pointed out that case reports generally receive fewer citations compared to original articles^17^. Despite receiving less citations, case reports still play a role in the realm of evidence-based medicine. Case reports provide details of one or a few cases for physicians to relate to their practice. Furthermore, these case reports are often interesting, and they also provide education value by highlighting rare diseases and potential or actual pitfalls that the clinicians can avoid if they encounter a similar case. Some journals have even created breakout journals dedicated to publishing case reports, as in the case of Malaysia Medical Association, where they established the Medical Journal of Malaysia Case Reports^18^.

MOJ has received article submissions from various sources. The top ten articles have been authored by individuals from seven different countries (Table II). Overall, the majority of corresponding authors originate from Malaysia, accounting for 102 of the published articles. This is expected as MOJ is the official journal of the Malaysian Orthopaedic Association (MOA), and all MOA members receive free copies of the journal. Besides that, half of the top ten countries with the most contributions are from South-East Asian countries. This is because every March issue of MOJ is designated as the ASEAN edition, serving as the official journal for ASEAN Orthopaedic Association (AOA) since 2010, following the discontinuation of the ASEAN Orthopaedic Journal^l2^. Despite receiving submissions from different countries, we have observed a lack of collaboration between countries. This is reflected in the low ratio of multiple-country publications to single-country publications. With the exception of three countries, all have 10% or less of their publications involving multiple-country collaborations. This underscores the importance of emphasising collaboration, as it provides networking opportunities and has the potential to increase the impact of research. Furthermore, international collaboration can enhance funding opportunities, particularly for developing countries^19^.

Keywords plays a crucial role in assisting researchers in finding relevant articles in the database^20^. By providing the right keywords, researchers can enhance the visibility and, consequently, the citation of the published articles in MOJ. By analysing the author’s keywords, trends in popular research published in MOJ can be traced. In a journal specialising in the field of orthopaedics, keywords such as “fracture”, “non-union”, “trauma”, “orthopaedics” and “reconstruction” are expected to be the most commonly used author’s keywords. Nevertheless, during the study period, something unique occurred as the world faced a once-in-a-lifetime pandemic^21,22^. During this challenging period, various articles related to both orthopaedics and the pandemic were produced, leading keywords such as “covid-19” and “pandemic” to become the top five most frequently used author’s keyword.

MOJ has adopted an open-access (OA) publishing policy, wherein accepted manuscripts are published online and are accessible to all the scholars^23^. In contrast to traditional subscription-based publishing model, scholars do not have to pay to read or share OA articles, resulting in more views per articles and, subsequently, higher citations per articles^23,24^. Generally, journals with OA publishing policy tend to receive more citations, as observed in various field, including hepatology, radiology, oncology and dermatology^23-26^.

There are several limitations present in this bibliometric analysis. Firstly, the data search was conducted exclusively using the Scopus database. While Scopus is one of the largest databases, this analysis is inherently limited because it excludes other databases such as Web of Science (now Clarivate Analytics) and Pubmed. Nevertheless, conducting the analysis with the Scopus database provides crucial information, such as article citations, which would not be available if the analysis were performed with a database like PubMed. Secondly, the analysis only covers the period during which MOJ was indexed in Scopus, and therefore, it excludes articles published prior to Scopus indexing (year 2007 to 2014).

## Conclusion

As MOJ continues to grow as a leading international journal, it is important to periodically review the journal’s progress. This bibliometric analysis offers insight into the contributions of various authors, institutions, countries and the trends in orthopaedic research. Despite the increasing citation counts and improvements in journal ranking, collaboration between different countries remains limited among the researchers in this region. It is hoped that this analysis can encourage more orthopaedic surgeons and researchers to collaborate and produce high-quality publications.
